# Cohort Profile: Effective Perinatal Intensive Care in Europe (EPICE) very preterm birth cohort

**DOI:** 10.1093/ije/dyz270

**Published:** 2020-02-07

**Authors:** Jennifer Zeitlin, Rolf F Maier, Marina Cuttini, Ulrika Aden, Klaus Boerch, Janusz Gadzinowski, Pierre-Henri Jarreau, Jo Lebeer, Mikael Norman, Pernille Pedersen, Stavros Petrou, Johanna M Pfeil, Liis Toome, Arno van Heijst, Patrick Van Reempts, Heili Varendi, Henrique Barros, Elizabeth S Draper, EPICE and SHIPS Research Group, J Lebeer, P Van Reempts, E Bruneel, E Cloet, A Oostra, E Ortibus, I Sarrechia, K Boerch, L Huusom, P Pedersen, T Weber, L Toome, H Varendi, M Männamaa, P Y Ancel, A Burguet, P H Jarreau, V Pierrat, P Truffert, R F Maier, M Zemlin, B Misselwitz, S Schmidt, L Wohlers, M Cuttini, D Di Lallo, G Ancora, D Baronciani, V Carnielli, I Croci, G Faldella, F Ferrari, F Franco, G Gargano, A van Heijst, C Koopman-Esseboom, J Gadzinowski, J Mazela, A Montgomery, T Pikuła, H Barros, R Costa, L Mendes Graça, M do Céu Machado, C Rodrigues, T Rodrigues, U Aden, A K Edstedt Bonamy, M Norman, E S Draper, E M Boyle, A Fenton, S J Johnson, B N Manktelow, D W A Milligan, S Mader, N Thiele, J M Walz, S Petrou, J Zeitlin, M Bonet, C Bonnet, R El Raffei, A Piedvache, A V Seppanen

**Affiliations:** 1 INSERM UMR 1153, Obstetrical, Perinatal and Pediatric Epidemiology Research Team (Epopé), Center for Epidemiology and Statistics Sorbonne Paris Cité, DHU Risks in pregnancy, Paris Descartes University, Paris, France; 2 Children's Hospital, University Hospital, Philipps University Marburg, Marburg, Germany; 3 Clinical Care and Management Innovation Research Area, Bambino Gesù Children’s Hospital, IRCCS, Rome, Italy; 4 Department of Womeńs and Childreńs Health, Karolinska Institutet, Stockholm, Sweden; 5 Department of Paediatrics, Hvidovre Hospital, Copenhagen University Hospital, Hvidovre, Denmark; 6 Department of Neonatology, Poznan University of Medical Sciences, Poznan, Poland; 7 Université Paris Descartes and Assistance Publique Hôpitaux de Paris, Hôpitaux Universitaire Paris Centre Site Cochin, DHU Risks in pregnancy, Service de Médecine et Réanimation néonatales de Port-Royal, Paris, France; 8 Department of Primary & Interdisciplinary Care, Disability Studies, Faculty of Medicine, University of Antwerp, Antwerp, Belgium; 9 Department of Clinical Science, Intervention and Technology, Division of Pediatrics, Karolinska Institutet, Stockholm, Sweden; 10 Department of Neonatal Medicine, Karolinska University Hospital, Stockholm, Sweden; 11 Department of Neonatology, Hvidovre Hospital, Hvidovre, Denmark; 12 The University of Warwick, Warwick Medical School (WMS), Coventry, UK; 13 University of Oxford, Nuffield Department of Primary Care Health Sciences, Oxford, UK; 14 European Foundation for the Care of Newborn Infants (EFCNI), Munich, Germany; 15 Tallinn Children’s Hospital, Tallinn, Estonia and University of Tartu, Tartu, Estonia; 16 Department of Neonatology, Radboud University Medical Center, Nijmegen, the Netherlands; 17 Department of Neonatology, Antwerp University Hospital, University of Antwerp, Antwerp, Belgium and Study Centre for Perinatal Epidemiology, Brussels, Belgium; 18 University of Tartu, Tartu University Hospital, Tartu, Estonia; 19 EPIUnit--Instituto de Saúde Pública da Universidade do Porto, Porto, Portugal; 20 Department of Health Sciences, University of Leicester, Leicester, UK

## Why was the cohort set up?

The Effective Perinatal Intensive Care in Europe (EPICE) cohort includes all births between 22 + 0 and 31 + 6 weeks of gestation in 2011/12 in 19 regions in 11 European countries. This cohort was set up to investigate the use of evidence-based interventions for prenatal and postnatal care of infants born very preterm (VPT) and to explore the associations between evidence-based care and their health and developmental outcomes. The first phase, ‘Effective perinatal intensive care in Europe’ (EPICE) focused on obstetric and neonatal care before and around the time of birth and during the neonatal hospitalization period, with follow-up at 2 years of corrected age (CA), while a second phase, ‘Screening for Health in Infants born very Preterm’ (SHIPS), assessed follow-up care provided in the first 5 years of life and neurodevelopmental outcomes at 5 years of age. Both phases were funded by the European Union [Seventh Framework Programme (FP7/2007–2013, No 259882; Horizon 2020 Research and Innovation Programme, No 633724].

Both phases are based on the premise that survival, neurodevelopmental outcome and health-related quality of life can be improved for children born VPT by promoting the use of evidence-based health care. Improving these outcomes is important as VPT birth, occurring in about 1–2% of births, constitutes one of the principal determinants of infant mortality and morbidity, accounting for up to 75% of neonatal deaths in 2015.^1^ Further, despite significant medical advances in survival over recent decades, after discharge from the neonatal intensive care unit (NICU), survivors of VPT birth remain at high risk of neurodevelopmental impairment, including cerebral palsy, cognitive impairment, visual and auditory deficits and behavioural problems. Several recent studies have found that rates of impairments are not decreasing over time.^2^^,^^3^

Countries in the European Union provide fertile ground for research comparing the care and outcomes of these babies. Despite having high national incomes, universal insurance or health coverage for pregnant women and newborns and widespread access to medical knowledge and care, >2-fold disparities exist in risk-adjusted VPT mortality and morbidity.^4^^,^^5^ These differences, which are also observed outside of Europe,^6–8^ strongly suggest that some of the variation in outcome relates to differences in obstetric and neonatal practices. This claim is supported by research within individual countries showing that practices in many obstetric and neonatal units are not based on the latest scientific evidence.^9–15^

The perinatal period is paramount for VPT infants, as care quality during this period impacts strongly on mortality and severe morbidity, but high-quality post-discharge care is also essential for infant and child development.^16^^,^^17^ Long-term adverse outcomes are related to neonatal morbidities, including brain lesions and respiratory morbidity and risks are higher with decreasing gestational age.^18–20^ However, prediction of outcomes is difficult and children with no identified neonatal morbidity may experience moderate or severe impairments and, conversely, children with neonatal morbidities may develop normally.^21^^,^^22^ Consequently, follow-up programmes aim to identify health problems early, enable interventions to improve outcome and to allow optimal management and coordination of health care. Despite the recognised importance of these programmes, little is known about their actual application and impact. As with perinatal care, there is a hypothesized wide variation in approaches to providing follow-up in Europe.

## Who is in the cohort?

**Figure 1.**
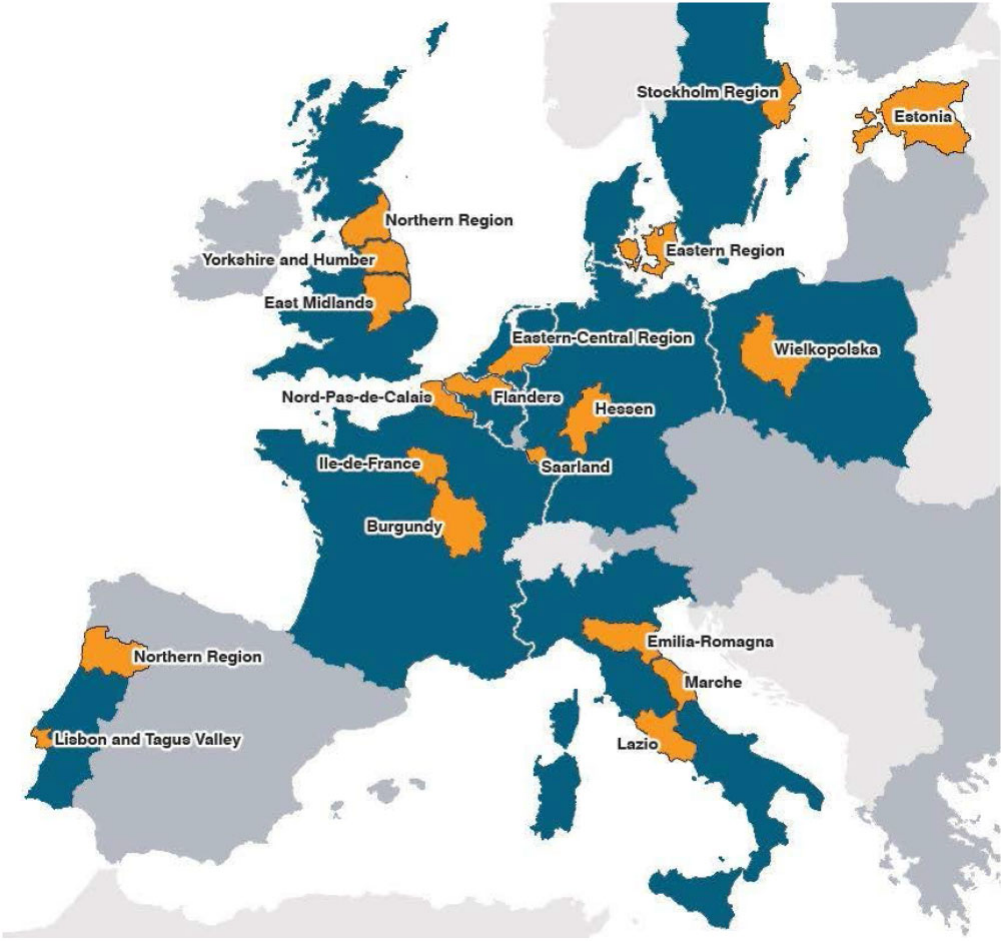
Regions included in the EPICE/SHIPS cohort: Flanders in Belgium; the Eastern Region of Denmark; Estonia (entire country); Burgundy, Ile-de-France and the Northern regions in France; Hesse and Saarland in Germany; Emilia- Romagna, Lazio and Marche regions in Italy; the Central and Eastern regions of The Netherlands; Wielkopolska in Poland; the Lisbon and Northern regions of Portugal; and the East Midlands, Northern and Yorkshire and Humber regions in the UK; and the Stockholm region in Sweden.

The EPICE cohort is a geographically defined study of stillbirths and live births from 22 + 0 to 31 + 6 weeks of gestation in 19 European regions (Fig. 1). Regions were selected with respect to geographic and organizational diversity and feasibility, meaning they had systems for collecting population data on VPT babies that could be modified to integrate the study protocol. In France, the EPICE study includes three regions of the national EPIPAGE 2 cohort study.^23^ Participating regions started data collection between March and July 2011 and the inclusion period lasted 12 months, except in France where it was 6 months. The study also collected information from the hospitals where these children were born and hospitalized. Questionnaires were sent to neonatal units with at least 10 VPT admissions and their associated maternity units in the spring of 2012.

Investigators abstracted data from medical records in obstetric and neonatal units for all births with a gestational age between 22 + 0 and 31 + 6 weeks. Gestational age was defined as the best obstetric assessment based on information on last menstrual period and antenatal ultrasounds, which are part of routine obstetric care in all regions. Inclusions were cross-checked against delivery ward registers or another external data source.

**Figure 2.**
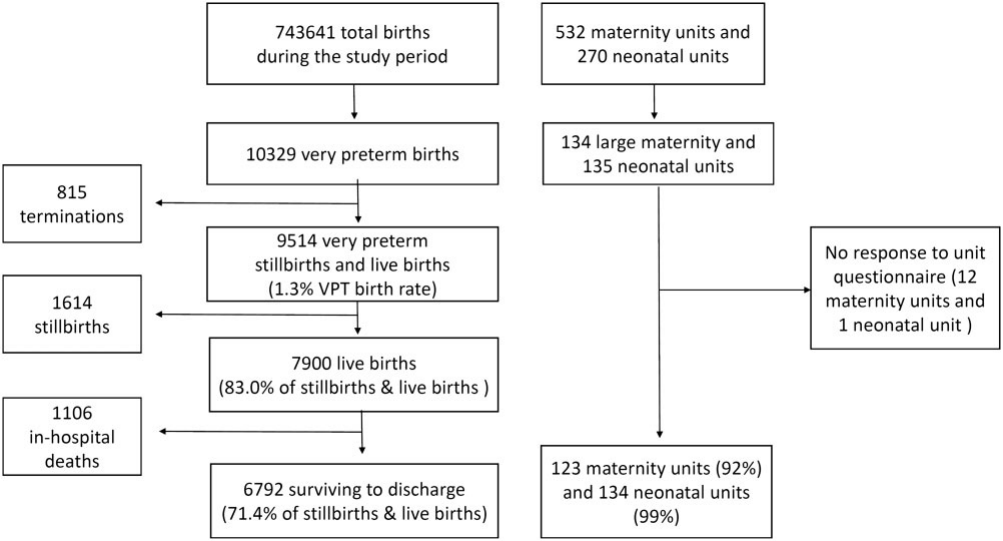
Inclusions in the EPICE very preterm birth cohort and maternity and neonatal unit study. Large units are neonatal units with ≥10 very preterm admissions per year and associated maternity units.

During the study period, 10 329 VPT births were included out of 743 641 total births, of which 815 were terminations of pregnancy and 9514 VPT stillbirths or live births (Fig. 2). Because the number of terminations reflect screening policies for congenital anomalies which differ greatly between countries,^24^ these are reported separately from calculations related to the baseline birth cohort.

The VPT birth rate for live births and stillbirths was 1.3%. Of all the VPT births, 7900 were live born (83.0%) and 6792 survived to discharge (71.4%). Regions in participating countries had VPT birth rates ranging from 0.9 to 1.5% (Table 1). The percentage of live born infants ranged from 77.8 to 88.9% and survival after live birth from 79.1 to 92.2%. Of the whole cohort of births, including stillbirths, the percentage discharged alive ranged from 63.6 to 78.8%. There were 532 maternity hospitals and 270 neonatal units in the EPICE regions. Of the 134 maternity units eligible for the unit study, 91.8% (123) responded to the questionnaire; 99.3% of eligible neonatal units (134/135) responded. Table 2 provides unit information by country.

**Table 1.** Inclusions in the cohort and selection related to very preterm birth rates and very preterm stillbirths and in-hospital deaths; TOP, termination of pregnancy

Country (regions)	Total births	VPT TOP	Total VPT births	VPT birth rate	VPT stillbirths	VPT live births	% live births	In-hospital deaths	Survivors to discharge	Survivors as a percent of live births	Survivors as a percent of total births
	*n*	*n*	*n*	%	*n*	*n*	%	*n*	*n*	%	%
Belgium (Flanders)	69 605	70	920	1.3	168	752	81.7	99	653	86.8	71.0
Denmark (Eastern region)	31 195	25	416	1.3	65	351	84.4	65	286	81.5	68.8
Germany (Hesse, Saarland)	56 582	(2)^a^	853	1.5	95	758	88.9	96	662	87.3	77.6
Estonia (whole country)	14 940	0	179	1.2	26	153	85.5	12	141	92.2	78.8
France (Burgundy, Northern region, Ile-de-France)^b^^,^^c^	129 341	367	1680	1.3	373	1307	77.8	198	1109	84.9	66.0
Italy (Emilia, Lazio, Marche)	109 444	(73)^d^	1326	1.2	192	1134	85.5	159	975	86.0	73.5
Netherlands (East-Central)	53 854	87	463	0.9	70	393	84.9	62	330	84.0	71.3
Poland (Wielkopolska)	38 246	7	393	1.0	77	316	80.4	66	250	79.1	63.6
Portugal (Lisbon, Northern)	62 847	95	879	1.4	155	724	82.4	117	607	83.8	69.1
Sweden (Stockholm region)	28 624	0	308	1.1	41	267	86.7	26	241	90.3	78.2
United Kingdom (East Midlands, Northern, Yorkshire)	148 963	89	2097	1.4	352	1745	83.2	206	1538	88.1	73.3
Total	743 641	815^a^^,^^d^	9514	1.3	1614	7900	83.0	1106^e^	6792	86.0	71.4

TOP, termination of pregnancy.

a Terminations could not be collected in Hesse, only terminations from Saarland are included.

b Inclusions over a 6 month period, so total births refer only to this period.

c 102 infants were not included because of parental refusals.

d Terminations could not be collected in Marche and Emilia, only terminations from Lazio are included.

e Two cases with no information on outcome are not included.

**Table 2.** Maternity and neonatal units included in the unit study

Country (regions)	Maternity units			Neonatal units		
	Total	Eligible^1^	Responded	Total	Eligible^a^	Responded
	*n*	*n*	*n*	*n*	*n*	*n*
Belgium (Flanders)	67	9	9	45	9	9
Denmark (Eastern region)	8	8	8	10	8	8
Germany (Hesse, Saarland)	66	14	13	21	14	14
Estonia (whole country)	21	3	2	7	4	4
France (Burgundy, Northern region, Ile-de-France)	147	22	22	48	23	23
Italy (Emilia, Lazio, Marche)	96	23	21	28	22	22
Netherlands (East-Central)	12	2	2	18	2	2
Poland (Wielkopolska)	36	4	4	13	4	4
Portugal (Lisbon, Northern)	30	17	17	23	17	17
Sweden (Stockholm region)	6	5	5	6	4	4
United Kingdom (East Midlands, Northern, Yorkshire)	43	27	20	51	28	27
Total	532	134	123	270	135	134

a Neonatal units with ≥10 very preterm admissions per year and associated maternity units.

Assessment of mortality-related selection is indispensable for research on VPT births. This is why it was crucial to include stillbirths and delivery room deaths in the cohort. All regions except for France received authorization to collect de-identified perinatal data on all VPT births to allow inclusion when it was not possible to contact parents, often the case for stillbirths and delivery room deaths. In France, 6% refused participation and minimal demographic and clinical data were collected on these births.

In all regions, inclusion in the follow-up required parental consent, and this was obtained at both follow-up waves. Each country team received ethical authorizations from local regional or hospital ethics boards, as required by national legislation. The European study was approved by the French Advisory Committee on Use of Health Data in Medical Research (CCTIRS, for EPICE) and the French Expert Committee for Research, Studies and Evaluations in the field of Health (CEREES for SHIPS) as well as the French National Commission for Data Protection and Liberties (CNIL) for both phases.

## How often have they been followed up?

Perinatal data were collected to discharge home from hospital or into long-term institutional care. At 2 years of CA, a parental questionnaire was used to collect information about socio-demographic characteristics, the child’s health and development and the child’s use of health care services. Between 5 and 6 years of age, follow-up was based on a parental questionnaire and, for children born at <28 weeks of gestation, a neurodevelopmental assessment. Plans for future follow-up aim for contact with families when the children are 11 or 12 years old.

**Figure 3:**
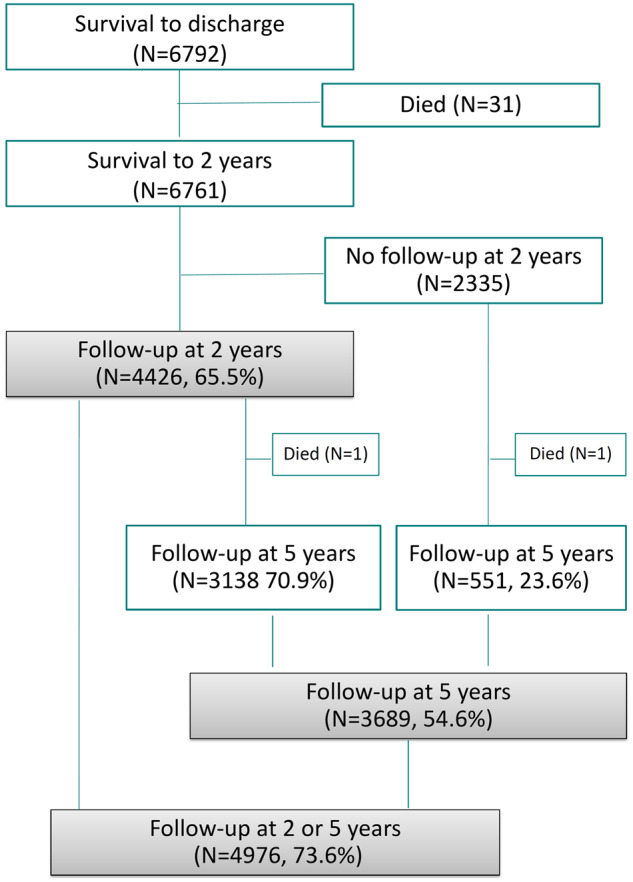
Follow-up of the EPICE cohort.

**Figure 4:**
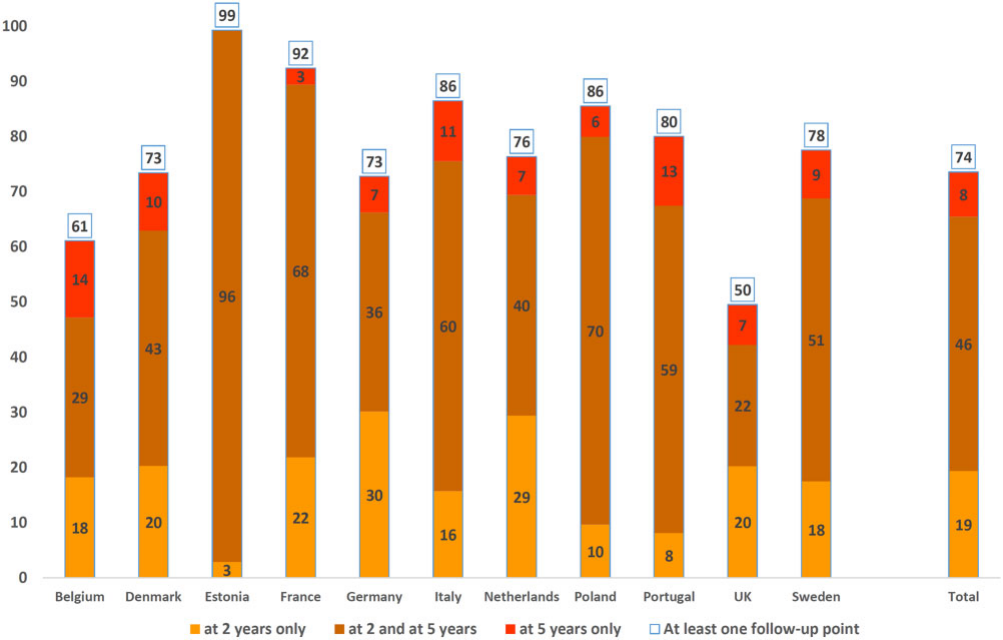
Follow-up rates at 2 and 5 years by country.

As shown in Fig. 3, 6761 children survived to 2 years of CA of which 65.5% were included in the 2-year follow-up, and 54.6% in the 5-year follow-up. At 5 years of age, 23.6% of the children who were not followed up at 2 years participated. This percentage was 70.9% for those followed up at 2 years. Therefore, at least one follow-up assessment was available for 73.6% of the cohort. The proportion of children followed up was higher among children born at <28 weeks of gestation who were invited for a neurodevelopmental assessment (61.2% followed at 5 years of age, 78.1% with at least one follow-up contact). Follow-up rates differed across country teams, with rates ranging from 42.2 to 99.3% at 2 years, 29.3 to 96.4% at 5 years and 49.5 to 99.3% for at least one follow-up contact (Fig. 4).

Table 3 displays socio-demographic and clinical data for the cohort surviving to 2 years of CA and those included in the study at 2 years of CA and at 5 years. Significance tests are provided overall and after adjustment for country because of differences in follow-up rates. In both follow-up waves, children born to younger, multiparous and migrant women were less likely to be included. Multiples were more likely to participate at 2, but not at 5 years. There were few differences in medical practices or morbidities, although bronchopulmonary dysplasia and transfer during neonatal care was higher among children who were not followed after adjustment for region at 5 years only. At 5 years, gestational age differed because of the use of two protocols that favoured a higher follow-up rate in children born before 28 weeks of gestational age. Children with a follow-up at 2 years, who were not followed-up at 5, had similar gross motor impairment and neurodevelopmental and language delays, but fewer mothers with lower education responded.

**Table 3.** Characteristics of cohort of infants surviving to 2 years and included in follow-up waves at 2 and 5 years; missing values are not included in calculation of percentages

	Survived to two years of corrected age (*n* = 6761)	Followed-up at 2 years of corrected age				Followed-up at 5 years of age			
		No *n*= 2335	Yes *n* = 4426	*P*-value	*P*-value^a^	No^b^*n* = 3070	Yes *n* = 3689	*P*-value	*P*-value^a^
	*n* (%)	*n* (%)	*n* (%)			*n* (%)	*n* (%)		
Maternal age (years)				<0.001	<0.001			<0.001	<0.001
<25	1123 (16.7)	573 (24.7)	550 (12.5)			682 (22.3)	441 (12.0)		
25–34	3821 (56.7)	1239 (53.4)	2582 (58.5)			1706 (55.9)	2113 (57.4)		
≥35	1791 (26.6)	508 (21.9)	1283 (29.1)			666 (21.8)	1125 (30.6)		
Missing	26 (0.4)	15 (0.6)	11 (0.3)			16 (0.5)	10 (0.3)		
Mother’s country of birth^c^				<0.001	<0.001			<0.001	<0.001
Foreign-born	1382 (20.4)	591 (27.1)	791 (18.8)			769 (26.8)	613 (17.4)		
Native-born	5001 (74.0)	1591 (72.9)	3410 (81.2)			2096 (73.2)	2903 (82.6)		
Missing	378 (5.6)	153 (6.6)	225 (5.1)			205 (6.7)	173 (4.7)		
Parity				<0.001	<0.001			<0.001	0.010
0	3812 (57.0)	1151 (49.9)	2661 (60.8)			1612 (53.0)	2199 (60.3)		
1	1649 (24.7)	589 (25.5)	1060 (24.2)			739 (24.3)	910 (25.0)		
≥2	1227 (18.4)	568 (24.6)	659 (15.0)			688 (22.6)	538 (14.8)		
Missing	73 (1.1)	27 (1.2)	46 (1.0)			31 (1.0)	42 (1.1)		
Type of pregnancy				0.0016	0.003			0.62	0.87
Singleton	4620 (68.3)	1653 (70.8)	2967 (67.0)			2107 (68.6)	2511 (68.1)		
Multiple	2141 (31.7)	682 (29.2)	1459 (33.0)			963 (31.4)	1178 (31.9)		
Missing	0 (0.0)	0 (0.0)	0 (0.0)			0 (0.0)	0 (0.0)		
Caesarean				0.027	0.99			0.026	0.86
No	2105 (31.4)	762 (33.1)	1343 (30.5)			997 (32.8)	1107 (30.2)		
Yes	4600 (68.6)	1538 (66.9)	3062 (69.5)			2045 (67.2)	2554 (69.8)		
Missing	56 (0.8)	35 (1.5)	21 (0.5)			28 (0.9)	28 (0.8)		
Gestational age (weeks)				0.003	0.085			<0.001	<0.001
23–25	544 (8.0)	204 (8.7)	339 (7.7)			219 (7.1)	323 (8.8)		
26–27	1113 (16.5)	333 (14.3)	780 (17.6)			414 (13.5)	698 (18.9)		
28–29	1823 (27.0)	638 (27.3)	1185 (26.8)			878 (28.6)	945 (25.6)		
30–31	3281 (48.5)	1160 (49.7)	2122 (47.9)			1559 (50.8)	1723 (46.7)		
Missing	0 (0.0)	0 (0.0)	0 (0.0)			0 (0.0)	0 (0.0)		
Sex				0.087	0.28			0.61	0.98
Boy	3630 (53.7)	1287 (55.1)	2344 (53.0)			1658 (54.0)	1970 (53.4)		
Girl	3130 (46.3)	1047 (44.9)	2082 (47.0)			1411 (46.0)	1723 (46.6)		
Missing	1 (0.1)	1 (0.1)	0 (0.0)			1 (0.1)	0 (0.0)		
SGA^d^				0.46	0.99			0.46	0.70
≥10	4572 (67.6)	1592 (68.2)	2980 (67.3)			2090 (68.1)	2481 (67.3)		
<10th percentile	2188 (32.4)	742 (31.8)	1446 (32.7)			979 (31.9)	1208 (32.7)		
Missing	1 (0.1)	1 (0.1)	0 (0.0)			1 (0.1)	0 (0.0)		
Severe neonatal morbidity^e^				0.21	0.56			0.85	0.95
No	5878 (89.5)	1989 (88.8)	3889 (89.8)			2648 (89.4)	3228 (89.5)		
Yes	693 (10.5)	251 (11.2)	442 (10.2)			315 (10.6)	378 (10.5)		
Missing	190 (2.8)	95 (4.1)	95 (2.2)			107 (3.5)	83 (2.3)		
BPD^f^				0.004	0.23			0.48	<0.001
No	5677 (86.0)	1920 (84.3)	3757 (86.9)			2571 (85.7)	3106 (86.3)		
Yes	926 (14.0)	358 (15.7)	568 (13.1)			430 (14.3)	494 (13.7)		
Missing	158 (2.3)	57 (2.4)	101 (2.3)			69 (2.3)	89 (2.4)		
Surgery				0.73	0.92			0.14	0.053
No	6051 (89.5)	2094 (89.7)	3957 (89.4)			2767 (90.1)	3284 (89.0)		
Yes	710 (10.5)	241 (10.3)	469 (10.6)			303 (9.9)	405 (11.0)		
Missing	0 (0.0)	0 (0.0)	0 (0.0)			0 (0.0)	0 (0.0)		
Transfer during neonatal care				0.033	0.098			0.29	<0.001
0	3955 (58.5)	1394 (59.7)	2561 (57.9)			1825 (59.4)	2129 (57.7)		
1	1866 (27.6)	600 (25.7)	1266 (28.6)			820 (26.7)	1046 (28.4)		
≥2	940 (13.9)	341 (14.6)	599 (13.5)			425 (13.8)	514 (13.9)		
Missing	0 (0.0)	0 (0.0)	0 (0.0)			0 (0.0)	0 (0.0)		
Breastfeeding at discharge				<0.001	<0.001			<0.001	<0.001
No	2767 (40.9)	1123 (50.6)	1644 (37.6)			1462 (49.3)	1304 (36.0)		
Yes	3821 (56.5)	1098 (49.4)	2723 (62.4)			1506 (50.7)	2314 (64.0)		
Missing	173 (2.6)	114 (4.9)	59 (1.3)			102 (3.3)	71 (1.9)		
From follow-up at 2 years corrected age						(*n*=1286)	(*n*=3138)		
Mother’s educational level								<0.001	<0.001
Lower secondary	–	–	–			310 (25.8)	528 (17.2)		
Upper secondary	–	–	–			463 (38.5)	1204 (39.3)		
Bachelor degree or less	–	–	–			261 (21.7)	755 (24.6)		
Master/Doctoral degree	–	–	–			169 (14.0)	578 (18.9)		
Missing						83 (6.5)	73 (2.3)		
Gross motor impairment								0.52	0.85
No	–	–	–			1187 (94.6)	2959 (95.1)		
Yes	–	–	–			68 (5.4)	154 (4.9)		
Missing						31 (2.4)	25 (0.8)		
Non-verbal cognitive impairment^g^								0.65	0.86
No	–	–	–			828 (85.0)	1999 (84.4)		
Yes	–	–	–			146 (15.0)	370 (15.6)		
Missing						71 (6.8)	24 (1.0)		
Expressive vocabulary <10 words								0.92	0.55
No	–	–	–			1073 (89.5)	2710 (89.4)		
Yes	–	–	–			126 (10.5)	322 (10.6)		
Missing						87 (6.8)	106 (3.4)		

a *P* value adjusted for country.

b Two children died between 2 and 5 years.

c Ethnicity in the UK.

d SGA, small for gestational age, based on intrauterine curves developed for the cohort.^25^

e Defined as intraventricular haemorrhage grades III–IV, cystic periventricular leukomalacia, surgical necrotizing enterocolitis, retinopathy of prematurity grades ≥3.

f BPD, Bronchopulmonary dysplasia defined as oxygen dependency or respiratory support at 36 weeks postmenstrual age.

g As a score <22 for non-verbal items on the Parent Report of Children's Abilities-Revised for preterm infants (PARCA-R), does not include children from the French regions where the PARCA-R was not used.

## What has been measured?

Table 4 provides a summary of the data collected in this cohort. Table 5 illustrates how these data sources contribute to the investigation of healthcare interventions and practices.

**Table 4.** Overview of data collected in the EPICE cohort

Period	Population	Type of data collection	Domains covered
Pregnancy, birth and the neonatal period until discharge home	All stillbirths and live births <32 weeks of gestational age	Medical chart abstraction in obstetric unit	Maternal characteristics Pregnancy complications Delivery
	Infants admitted to a neonatal unit	Medical chart abstraction in neonatal units	Neonatal course and morbidity Medical interventions Feeding and growth
	Obstetric and neonatal units where babies were born and/or hospitalized^a^	Questionnaire to heads of maternity and neonatal units	Structural variables: volume of births or admissions, staffing, services Policies and practices: written protocols, practices, including ethics and follow-up
	Units in 6 regions	Qualitative survey	Triggers, facilitators and barriers for change in policies or guidelines in neonatal units
	Countries and regions	National/regional case studies	Policies/recommendations related to care of very preterm infants
2 years corrected age	Surviving children with parents consenting to follow-up	Parent report questionnaire	Parent reported health problems Growth (weight, height, head circumference) Parent Report of Children's Abilities - Revised for preterm infants (PARCA-R) Healthcare utilization Socio-economic variables
5 years of age	Surviving children with parents consenting to follow-up (in 19 regions)	Parent report questionnaire	Parent reported health problems and diagnoses Growth (weight, height) Modified Ages and Stages (ASQ) parental report developmental assessment Healthcare utilization Healthcare expenses Strengths and difficulties questionnaire (SDQ): parent-rated assessment of behaviour, attention, peer relationship, emotional problems and pro-social behaviour Quality of life (PedsQL): a parent proxy report for the health related quality of life of children aged 5–6 years Parental wellbeing (MF5) a validated subscale recommended for screening of mood and anxiety disorders derived from short form 36. Socio-economic variables
	Surviving children born <28 weeks of GA with parents consenting to follow-up	Neurodevelopmental assessments by a clinical psychologist	Clinical assessments: (1) general cognition (Wechsler Preschool and Primary Scale of Intelligence, 4th Edition, WPPSI-IV^26^ WPPSI-III, WPPSI-R); (2) Working memory (WPPSI-IV Working memory Index or Clinical Evaluation of Language Fundamentals (CELF-4-NL); (3) Processing speed (WPPSI-IV Processing speed index or WPPSI-R); (4) Visual-spatial processing (Developmental Neuropsycholgical Test 2nd Edition (NEPSY-II)^27^ Arrows and design copying); (5) Motor performance [Movement Assessment Battery for Children 2nd Edition (MABC-2)^28^]. Non-verbal IQ [Raven’s Coloured Progressive Matrices^29^ or Snijders-Oomen Nonverbal Intelligence Test (SON-R).]
	Random selection of surviving children born <28 weeks in 5 regions (8–9 with impairments, 2–4 without)	Qualitative survey	Parental experiences with follow-up care
	Countries and regions	Questionnaire to research team, collecting data from multiple sources	Existence and content of national or regional follow-up programmes or recommendations

a Only neonatal units with ≥10 very preterm admissions per year and associated maternity units.

**Table 5.** Information on healthcare and follow-up policies and practices

Data on policies and practices	Cohort study	Obstetric unit study	Neonatal unit study	Governance/ Policy studies	Qualitative studies
Evidence-based interventions					
Delivery in tertiary centres	X	X		X	
Antibiotics for preterm premature rupture of membranes (PPROM)		X		X	
Tocolysis		X		X	
Administration of antenatal corticosteroids	X	X		X	
Magnesium sulfate for neuroprotection	X	X		X	
Delivery by caesarean section	X	X		X	
Time (early or late) for cord clamping		X		X	
Management of hypothermia	X	X	X	X	
Surfactant replacement therapy	X		X	X	
Inhaled nitric oxide	X		X	X	
Breastfeeding	X	X	X	X	
Management of patent ductus arteriosus	X		X	X	
Developmental care/skin-to-skin care			X	X	
Bronchopulmonary dysplasia (BPD) Prevention strategies (vitamin A/caffeine)			X	X	
Postnatal corticosteroids (non-use)	X		X	X	
Retinopathy of prematurity (ROP) screening and treatment	X		X	X	
Ethics and active management <24 weeks gestational age					
Written recommendations/guidelines			X	X	
Decisions to use caesarean section	X	X			
Presence of neonatologists		X	X		
Involvement of parents in decisions			X		
Withholding and withdrawing care	X	X	X		
Evidence-based protocols and procedures					
Meetings and review of literature		X	X		
Participation in research		X	X		
Data collection, evaluation and audit		X	X		X
Process for adopting change					X
Follow-up after discharge home					
Existence of follow-up programme	X		X	X	
Target population for follow-up			X	X	
Follow-up schedule	X		X	X	
Follow-up content			X	X	
Uptake and experiences of follow-up	X		X	X	X

Perinatal data on maternal characteristics, pregnancy complications, birth and the neonatal course were abstracted from medical records in obstetric and neonatal units using pretested standardized questionnaires with common definitions based on a previous study.^5^

At 2 years of corrected age, parents filled in a questionnaire on health, neurodevelopmental outcomes, growth, health service use and socio-demographic information. The questionnaire was developed in English and translated into national languages, back-translated and pretested by the country teams. The questionnaire included a validated developmental assessment tool, the Parent Report of Children's Abilities - Revised for preterm infants (PARCA-R), which includes the MacArthur language assessment short form.^30^^,^^31^ We also developed four questions on language acquisition for countries where the MacArthur was not available. In the French region, the Ages and Stages questionnaire was used instead as this instrument was validated in France whereas the PARCA-R was not.^32^

At 5 years, the Health and Wellbeing study used a parent-report questionnaire to assess health and child development, health service use, healthcare related costs, satisfaction with healthcare services, family wellbeing and socio-demographics. It assessed the child’s development using the communication and problem-solving skills domains of the Ages and Stages Questionnaire (ASQ-3) 72 months, as well as other validated instruments previously used in preterm populations: the Strengths and Difficulties Questionnaire (SDQ),^33–35^ the Pediatric Quality of Life Inventory (PedsQL);^36^ and for parents, the Five Item Mental Health Inventory (MHI-5).^37^^,^^38^ The questionnaire also included four forced choice items to assess neurosensory impairments (vision, hearing, fine and gross motor skills), based on standard definitions for classifying neurodevelopmental disability in preterm populations, adapted for children at 5 years of age.^30^ The questionnaire was piloted in English with parents recruited through the European Foundation for the Care of Newborn Infants (EFCNI). Final translated versions were pretested by parents with 5 year old children.

The Neurodevelopmental Assessment Study carried out neurodevelopmental assessments at 5 years of age for children born before 28 weeks of gestation. A preferred test battery was defined for five domains: general cognition, working memory, processing speed, visual-spatial processing and motor performance. If the preferred test was not nationally normed, countries substituted equivalent tests, as listed in Table 4. The test battery also included a non-verbal IQ test for children unable to complete the Wechsler Preschool and Primary Scale of Intelligence (WPPSI) assessment.

## Contextual data

The maternity and neonatal unit study collected information on structural characteristics (level of specialization, personnel, activity levels), protocols and practices related to selected medical interventions, ethical decisions, decision-making processes and the structure and content of follow-up programmes using a questionnaire sent to the head of the unit in the spring of 2012. The questions were formulated so that they could be answered in the same way by different members of staff. Questionnaires were pretested outside the study regions in all countries and then translated and back translated in France and in Italy (for maternity units only). They were administered in English in the other countries.

The qualitative study in neonatal units investigated decision-making processes in neonatal intensive care units for the development, implementation and evaluation of clinical policies and/or guidelines, based on the current policies in use on the unit. It was carried out in two randomly selected tertiary NICUs in regions from Denmark, France, Germany, Italy, Portugal and the UK in 2012. Two physicians and two nurses were interviewed in each participating unit for a total of 44 in-depth face-to-face personal interviews.

The qualitative study with parents focused on the use and uptake of follow-up care and families’ experiences in order to identify the key factors perceived by parents as barriers and facilitators for participation in clinical follow-up. The study used in-depth semi-structured personal interviews. The study population included parents having children with and without severe impairments at 5 years of age in regions in Italy, Belgium, France, Germany and Portugal (62 total interviews, 42 for children with impairments and 20 for children without impairments).

### Contextual data on governance and policy

In both project phases, data were collected to describe European, national and regional laws and recommendations affecting the use of evidence-based practices (EPICE) and follow-up programmes (SHIPS) for VPT infants. Standardized structured questionnaires were completed by participating teams at the start of each project phase to compile information from their country, including references and information about governmental and non-governmental regulatory structures. In the questionnaire on follow-up programmes, data included information on the start date, structural organization, funding, target populations, content, duration, providers, referral services, family satisfaction and evaluation of implementation.

## What has it found? Key findings and publications

- Mortality at birth and during the neonatal hospitalization after VPT birth varies widely across Europe.^39^ This variability extends to severe neonatal morbidity, which we were able to measure using standardized definitions that are not available in most other studies.^39^ Continued variability between European regions was observed in the neurodevelopmental outcomes of these children at 2 years of CA.^40^
- Differences in ethical decision-making contribute to survival variation across European countries.^41^^,^^42^ However, these survival differences do not appear to impact strongly on severe morbidity rates.^39^
- There is large between-country variability in use of obstetric and neonatal interventions that is independent of patient case-mix.^43–46^ This includes use of non-evidence-based interventions, and non-use of interventions well proven to be effective at lowering mortality and morbidity. We found that 41.7% of babies did not receive a combination of four basic evidence-based interventions and that this was associated with significant excess mortality and morbidity.^47^
- Evaluations of some practices confirmed previous findings and provided new evidence of their effectiveness, including antenatal steroids which reduced mortality even when administered close to delivery, and liberal parental visiting policies which positively impacted exclusive breastfeeding.^48^^,^^49^
- Some practices with a long-term evidence base were confirmed to markedly influence mortality and morbidity, for example, prevention of hypothermia.^46^^,^^50^
- Some interventions have not yet been assessed for their effectiveness because their impact can only be measured later in childhood, such as postnatal steroids and breastfeeding,^44^^,^^51^ but we found high practice variability unexplained by case-mix. Some highly variable practice patterns reflect unsettled science, such as use of caesarean section for breech deliveries.^52^ In this example, our research suggests that large protective benefits found in previous studies likely reflect confounding and the question of what constitutes best practice remains open.
- Neonatal unit practices continue to exert an influence after discharge, as shown by the negative impact of multiple neonatal transfers and mixed-feeding (formula and breastmilk) on breastfeeding continuation to 6 months.^53^
- After discharge, parent reported healthcare use in the first 2 years differs greatly between countries with from 53.7 to 100% of children having at least one specialist consultation.^54^ Use of specialist care was related to perinatal risk factors, but not explained by them. In some countries, children whose mothers had lower educational attainment received fewer specialist services.

## What are the main strengths and weaknesses?

The cohort’s main strengths are the large population-based sample from a diverse set of regions across Europe and the availability of information on policies and practices to investigate care and health and developmental outcomes. This diversity is a gauge of the generalizability of study findings. Another strength is that we included live births and stillbirths starting at the gestational age cut-off recommended by the World Health Organization, independently from national registration rules; this allowed us to obtain comparable data on survival across regions. Additionally, we analysed labour ward deaths to develop reliable information on the outcome of all VPT live births and investigate policy differences with respect to admission for neonatal care. Other strengths are the larger sample sizes obtained by including multiple regions, having standardized and pretested protocols building on previous European research, and cross-checking inclusions to confirm completeness.

The main challenge is managing loss to follow-up, including regional differences in loss to follow-up, particularly where this may relate to the child’s health difficulties or developmental impairments. Furthermore, although the sample is large, the number of children in individual hospitals was more limited, constraining our ability to carry out analyses at the hospital-level. Finally, the diversity of health care provision can make it difficult to define comparable exposures for assessment of effectiveness.

## Can I get hold of the data? Where can I find out more?

More information can be found on the EPICE/SHIPS website (www.epiceproject.eu). The EPICE cohort is working with the RECAP Preterm consortium of VPT cohorts (recap-preterm.eu) to establish a platform to catalogue these studies and make them available to the wider community of researchers. This project will be completed in March 2021. In the meantime, requests for collaboration can be sent to the EPICE/SHIPS cohort coordinator using the contact form on the project website (corresponding author). The consortium has a process for initiating new analyses that involves submitting a protocol for approval by regional teams and then creating a writing group with interested members of the EPICE/SHIPS research group.

**Profile in a nutshell**

- The EPICE cohort is an area-based multinational cohort of VPT children that aims to investigate the use and consequences of evidence-based health care.
- All births between 22 + 0 and 31 + 6 weeks of gestation were included in 19 regions in 11 European countries over a 12 month period in 2011/12 (*n* = 10 329). Data were also collected from maternity and neonatal units and on regional healthcare policies.
- Follow-up of 6792 survivors to discharge home from hospital included parent-report questionnaires at 2 and 5 years of age and a clinical assessment at 5 years for a sub-set of children born before 28 weeks of gestation. 74% of the cohort participated in at least one follow-up.
- The dataset includes information on the pregnancy, birth and neonatal hospitalization, abstracted from medical records; parent-report measures of healthcare use, health needs, quality of life and neurodevelopment, based on validated instruments; clinical assessments of cognitive and motor function at 5 years; information on the structural characteristics of hospitals and their policies on use of 17 medical interventions as well as follow-up programmes in the units and regions.
- EPICE is part of the RECAP Preterm project which is setting up a research platform for European VPT cohorts.

## EPICE and SHIPS Research Group

Belgium (J Lebeer, P Van Reempts, E Bruneel, E Cloet, A Oostra, E Ortibus, I Sarrechia); Denmark (K Boerch, L Huusom, P Pedersen, T Weber); Estonia (L Toome, H Varendi, M Männamaa); France (PY Ancel, A Burguet, PH Jarreau, V Pierrat, P Truffert); Germany (RF Maier, M Zemlin, B Misselwitz, S Schmidt, L Wohlers,) Italy (M Cuttini, D Di Lallo, G Ancora, D Baronciani, V Carnielli, I Croci, G Faldella, F Ferrari, F Franco, G Gargano); The Netherlands (A van Heijst, C Koopman-Esseboom); Poland (J Gadzinowski, J Mazela, A Montgomery, T Pikuła) Portugal (H Barros, R Costa, L Mendes Graça, M do Céu Machado, C Rodrigues, T Rodrigues); Sweden (U Aden, AK Edstedt Bonamy, M Norman); United Kingdom (ES Draper, EM Boyle, A Fenton, SJ Johnson, BN Manktelow, DWA Milligan) EFCNI (S Mader, N Thiele, JM Walz); Health Economics team (S Petrou); Inserm Coordination (J Zeitlin, M Bonet, C Bonnet, R El Raffei, A Piedvache, AV Seppanen).

## Supplementary data

Supplementary data are available at *IJE* online.

## Supplementary Material

dyz270_Supplementary_Data
