# Supplementary material for: Cohort Profile: Effective Perinatal Intensive Care in Europe (EPICE) very preterm birth cohort
Source: Int J Epidemiol. 2020 Feb 7;49(2):372–86. doi: 10.1093/ije/dyz270 (PMC7266542; doi:10.1093/ije/dyz270)
Supplement: dyz270_Supplementary_Data [file dyz270_supplementary_data.docx]

**Acknowledgements**

We would like to acknowledge the participation of regional psychologists involved in the clinical assessments of children in the SHIPS cohort at five years of age, the parent associations participating in our parental advisory board, further funding within individual regions for the EPICE study, and the Departments of Obstetrics and Neonatology from the hospitals in the participating regions.

Acknowledgements for psychologists for the clinical assessment: Italy: Elena Arpi Chiara Giorno, Alessandra Montesi, Stefania Pezzotti, Alessandra Sansavini, Silvia Savini. Belgium: Lieve Baeyens, Charlotte Cleuren, Anke De Wel, Kristel Liesens, Frédérique Philippeth, Inge Schramme

Institutions participating in the parents’ advisory board

Bliss, United Kingdom, Fundacja Wcześniak Rodzice-Rodzicom, Poland Föräldraföreningen för Prematurfödda Barn, Sweden, Ilitominon, Greece, Irish Neonatal Health Alliance (INHA), Ireland, Melletted a helyem – Right(s) beside you, Hungary, MTÜ Enneagsed lapsed, Estonia, Piccino Picciò Onlus/Vivere Onlus, Italy, Prematura, Spain, SOS Préma, France, Vereniging van Ouders van Couveusekinderen, Netherlands, Vlaamse Vereniging voor Ouders van Couveusekinderen (VVOC), Belgium, XXS – Associação Portuguesa de Apoio ao Bebé Prematuro, Portugal, Bundesverband "Das frühgeborene Kind", Germany.

Additional regional support

Additional funding was received for the EPICE project in the following regions: France (French Institute of Public Health Research/Institute of Public Health and its partners the French Health Ministry, the National Institute of Health and Medical Research, the National Institute of Cancer, and the National Solidarity Fund for Autonomy; grant ANR-11-EQPX-0038 from the National Research Agency through the French Equipex Program of Investments in the Future; and the PremUp Foundation); Poland (2012-2015 allocation of funds for international projects from the Polish Ministry of Science and Higher Education); Portugal (by FEDER through the Operational Programme Competitiveness and Internationalization and national funding from the Foundation for Science and Technology – FCT (Portuguese Ministry of Science, Technology and Higher Education), under the Unidade de Investigação em Epidemiologia - Instituto de Saúde Pública da Universidade do Porto); UK (funding for The Neonatal Survey from Neonatal Networks for East Midlands and Yorkshire & Humber regions).

Hospitals participating in the EPICE/SHIPS cohorts

**Belgium (Flanders):** ASZ Campus Geraardsbergen, Geraardsbergen; AZ Sint Maarten, Campus Zwartzustervest, Mechelen; AZ Sint Lucas, Assebroek; AZ Heilige Familie, Rumst-Reet; Sint Jozefskliniek, Izegem; AZ Sint Jozef, Malle; Sint Augustinus - MISA, Wilrijk; Onze Lieve Vrouwziekenhuis Campus Asse, Asse; AZ Diest, Diest; AZ Zeno Campus Knokke - Heist, Knokke-Heist; AZ Groeninge, Kortrijk; Sint Jozefkliniek – Campus Bornem, Bornem; Sint Vincentiusziekenhuis, Deinze; Maria Middelares, Gent; AZ Oudenaarde, Oudenaarde; AZ Glorieux, Ronse; AZ Delta Campus Menen, Menen; AZ Sint Elisabeth, Zottegem; ZOL – Campus Sint Jan, Genk; Jessa Ziekenhuis Campus Virga Jesse, Hasselt; Sint Franciskusziekenhuis, Heusden-Zolder; Maria Ziekenhuis Noord-Limburg, Overpelt; Sint Trudoziekenhuis, Sint-Truiden; AZ Damiaan, Oostende; AZ Sint Lucas, Gent; AZ Sint Blasius, Dendermonde; AZ Delta Campus Wilgenstraat, Roeselare; UZ Brussel, Brussel; ZNA Jan Palfijn, Merksem; Sint Andriesziekenhuis, Tielt; ZNA Middelheim, Antwerpen; Imeldaziekenhuis, Bonheiden; AZ Sint Maarten – Campus Duffel, Duffel; AZ KLINA, Brasschaat; AZ Jan Portaels, Vilvoorde; Universitair Ziekenhuis Antwerpen, Edegem; UZ Leuven Campus Gasthuisberg, Leuven; UZ Gent, Gent; Sint Vincentiusziekenhuis – Campus Sint Jozef, Mortsel; AZ Alma, Eeklo; AZ Turnhout, Turnhout; Heilig Hartziekenhuis, Mol; AZ Sint Jan Campus Henri Serruys, Oostende; Ziekenhuis Maas & Kempen, Bree; Sint Vincentius Ziekenhuis, Antwerpen; AZ Vesalius – Campus Sint Jacobus, Tongeren; ASZ – Campus Aalst, Aalst; Onze Lieve Vrouwziekenhuis – Campus Aalst, Aalst; AZ Delta Campus Stedelijk Ziekenhuis, Roeselare; AZ Sint Rembert, Torhout; AZ Monica – Campus Deurne, Deurne; AZ Lokeren, Lokeren; Jan Ypermanziekenhuis, Ieper; AZ Sint Elisabeth, Herentals; AZ Sint Jan, Brugge; AZ Jan Palfijn, Gent; AZ Sint Augustinus Veurne, Veurne; RZ Sint Maria, Halle; Heilig Hart Ziekenhuis, Leuven; AZ Nikolaas – Campus SM, Sint-Niklaas; Onze Lieve Vrouw van Lourdes Ziekenhuis Waregem vzw, Waregem; ACZA Ziekenhuis – Campus Sint Erasmus, Borgerhout; Heilig Hart, Lier; AZ Sint Dimpna, Geel; Heilig Hart, Tienen.

**Estonia:** Tallinn Children’s Hospital, Unit of Newborns and Infants; Tallinna Children’s Hospital, Paediatric Intensive Care Unit; Tartu University Hospital, Neonatal Unit; Tartu University Hospital, Paediatric Intensive Care Unit; East-Tallinn Central Hospital, Neonatal Unit; West-Tallinn Central Hospital, Neonatal Unit.

**Denmark (Eastern Region):** University Hospital of Copenhagen (Rigshospitalet); Hvidovre University Hospital; Herlev University Hospital; Hilleroed University Hospital; Roskilde University Hospital; Holbaek University Hospital; Naestved University Hospital; Nykoebing Falster Sygehus; University Hospital of Southern Denmark

**France (Burgundy):** CH d'Autun, Autun; CH Auxerre, Auxerre; CH de Beaune, Beaune; CH William Morey, Chalon Sur Saone; Clinique de Cosne-Sur-Loire, Cosne-Sur-Loire; CH de Decize, Decize; CHU Le Bocage - Hôpital D'enfants, Dijon; Clinique Sainte-Marthe, Dijon; Site Hospitalier Foch, Le Creusot; CH Les Chanaux, Macon; CH de Nevers, Nevers; CH Les Charmes, Paray Le Monial; CH de Semur-En-Auxois, Semur En Auxois; CH de Sens, Sens;

**France (Ile-de-France**): Hôpital Prive D'Antony, Antony; CH Victor Dupouy, Argenteuil; CH Arpajon, Arpajon; Hôpital Prive D’Athis-Mons, Athis-Mons; Hôpital Européen La Roseraie, Aubervilliers; CHI Robert Ballanger, Aulnay Sous-Bois; CH Intercommunal Des Portes de L’Oise, Beaumont Sur Oise; CHU Jean Verdier, Bondy; Clinique Ambroise Paré, Bourg-La-Reine; Hôpital Privé de Marne Chantereine, Brou Sur Chantereine; Hôpital Prive de Marne La Vallée, Bry Sur Marne; Hôpital Saint-Camille, Bry Sur Marne; Clinique de Champigny-Hôpital Paul D'Egine, Champigny Sur Marne; Hôpital Antoine Béclère, Clamart; Hôpital Beaujon, Clichy; CH Louis Mourier, Colombes; Clinique Du Parisis, Cormeilles-En-Parisis; CH René Arbeltier, Coulommiers; CHI Créteil, Créteil; Clinique Claude Bernard, Ermont; CH Sud Essonne, Etampes - Dourdan; CH Louise Michel, Evry; Clinique de L'Essonne Evry, Evry; CMO D'Evry, Evry; Polyclinique de La Forêt, Fontainebleau; CH Fontainebleau, Fontainebleau; CH de Gonesse, Gonesse; Clinique Lambert, La Garenne-Colombes; CHG de Marne La Vallée, Lagny; Hôpital Prive de Seine-Saint-Denis, Le Blanc Mesnil; CH de Versailles-André Mignot, Le Chesnay; Hôpital Prive de Parly 2 Le Chesnay, Le Chesnay; CHU Kremlin Bicêtre, Le Kremlin-Bicêtre; Maternité Des Lilas, Les Lilas; Institut Hospitalier Franco-Britannique, Levallois Perret; Clinique Conti, L’Isle-Adam; Polyclinique Vauban, Livry Gargan; Clinique de L'Yvette, Longjumeau; CH de Longjumeau, Longjumeau; CH Mantes La Jolie, Mantes La Jolie; Hôpital Prive Jacques Cartier, Massy; CH de Meaux, Meaux; CH Marc Jacquet, Melun; Polyclinique Saint Jean, Melun; Clinique de Meudon La Foret, Meudon La Foret; CH Intercommunal de Meulan-Les Mureaux, Meulan-En-Yvelines; CH de Montereau, Montereau; CHI Le Raincy-Montfermeil, Montfermeil; Groupe Hospitalier Eaubonne-Montmorency, Montmorency; CHI André Grégoire, Montreuil; Hôpital Max Fourestier, Nanterre; CH Neuilly Courbevoie, Neuilly Sur Seine; Hôpital Américain, Neuilly Sur Seine; Clinique Sainte-Isabelle, Neuilly-Sur-Seine; Hôpital Prive Armand Brillard, Nogent Sur Marne; CH D’Orsay, Orsay; Clinique de La Muette, Paris; Clinique Jeanne D'arc, Paris; Clinique Leonard de Vinci, Paris; Clinique Sainte Thérèse, Paris; Clinique Saint-Louis, Paris; GH Armand Trousseau - La Roche-Guyon, Paris; GH Diaconesses Croix St Simon, Paris; GH Pitié-Salpêtrière, Paris; GH Saint Joseph / Notre Dame de Bon-Secours, Paris; GIH Bichat/ Claude Bernard, Paris; Hôpital Cochin-Port Royal, Paris; Hôpital Lariboisière, Paris; Hôpital Les Bluets, Paris; Hôpital Necker, Paris; Hôpital Robert Debré, Paris; Hôpital Saint-Antoine, Paris; Hôpital Tenon, Paris; Institut de Puériculture Et de Périnatalogie, Paris; Institut Mutualiste Montsouris, Paris; Maternité Sainte-Félicité, Paris; CHI Poissy/Saint-Germain-En-Laye, Poissy; CH René Dubos, Pontoise; Centre Hospitalier Léon Binet, Provins; Hôpital Prive Claude Galien, Quincy-Sous-Sénart; CH de Rambouillet, Rambouillet; Clinique Les Martinets, Rueil-Malmaison; CH Des Quatre Villes, Saint Cloud; CH de Saint Denis, Saint Denis; Hôpital Esquirol St Maurice, Saint Maurice; Clinique Saint Germain, Saint-Germain-En-Laye; Hôpital Prive Nord Parisien, Sarcelles; CH Des Quatre Villes, Sèvres; Hôpital Militaire Begin, St Mande; Clinique Gaston Métivet, St Maur Des Fosses; Clinique de L'Estrées, Stains; Hôpital Foch, Suresnes; Clinique de Tournan, Tournan-En-Brie; Hôpital Prive de L'Ouest Parisien, Trappes; Clinique Du Vert-Galant, Tremblay En France; Hôpital Prive de Versailles- Franciscaines, Versailles; CH Intercommunal, Villeneuve Saint Georges; Clinique Les Noriets, Vitry Sur Seine;

**France (Northern Region**): CH D'Armentières, Armentières; CH D’Arras, Arras; Clinique Bon-Secours, Arras; CH de Béthune, Béthune; Clinique Anne D’Artois, Béthune; CH de Boulogne-Sur-Mer, Boulogne Sur Mer; CH de Calais, Calais; CH de Cambrai, Cambrai; Polyclinique Sainte-Marie, Cambrai; CH de Denain, Denain; Polyclinique de La Clarence, Divion; CH de Douai, Douai; Polyclinique Villette, Dunkerque; CH de Fourmies, Fourmies; GCS Flandre Maritime, Grande Synthe; CH D'hazebrouck, Hazebrouck; CH de La Région de St Omer, Helfaut; Clinique Saint-Amé, Lambres-Les-Douai; CH du Cateau, Le Cateau Cambrésis; CH de Lens, Lens; Polyclinique de Riaumont de Lievin, Lievin; GHI CL St-Vincent de Paul, Lille; Hôpital Jeanne de Flandre, Lille; Pavillon Du Bois, Lille; CH Sambre-Avesnois, Maubeuge; Polyclinique Du Val de Sambre, Maubeuge; CHAM Site Principal, Rang Du Fliers; Maternité Paul Gellé, Roubaix; CH de Seclin, Seclin; Centre Médical Chirurgical Obstétrical Côte d'Opale, St Martin / Boulogne; Clinique Maternité du Parc, St Saulve; Clinique Du Val de Lys, Tourcoing; Hôpital Guy Chatiliez, Tourcoing; CH de Valenciennes, Valenciennes; Nouvelle Clinique Villeneuve D’ Ascq, Villeneuve d'Ascq.

**Germany (Hesse and Saarland):** Klinik für Kinder- und Jugendmedizin & Klinik für Frauenheilkunde und Geburtshilfe, Klinikum Bad Hersfeld, Bad Hersfeld; Neonatologie, Darmstaedter Kinderkliniken & Frauenklinik, Klinikum Darmstadt, Darmstadt; Klinik für Neonatologie & Frauenklinik, Buergerhospital, Frankfurt; Klinik für Kinder- und Jugendmedizin & Klinik für Gynaekologie und Geburtshilfe, Klinikum Frankfurt Hoechst, Frankfurt; Zentrum für Kinderheilkunde & Klinik fuer Frauenheilkunde und Geburtshilfe, Universitaetsklinikum Frankfurt, Frankfurt; Klinik für Kinder- und Jugendmedizin & Frauenklinik, Klinikum Fulda, Fulda; Klinik für Kinder- und Jugendmedizin & Frauenklinik, Main-Kinzig-Kliniken, Gelnhausen; Allgemeine Paediatrie und Neonatologie & Zentrum für Frauenheilkunde und Geburtshilfe, Universitaetsklinikum Giessen, Giessen; Klinik für Kinder- und Jugendmedizin & Klinik für Gynaekologie und Geburtshilfe, Klinikum Hanau, Hanau; Kinderklinik & Klinik für Frauenheilkunde und Geburtshilfe, Klinikum Kassel, Kassel; Klinik für Kinder- und Jugendmedizin & Klinik für Frauenheilkunde und Geburtshilfe, Universitaetsklinikum Marburg, Marburg; Klinik für Kinder- und Jugendmedizin & Klinik fuer Gynaekologie und Geburtshilfe, Sana Klinikum Offenbach, Offenbach; Klinik für Kinder- und Jugendmedizin & Frauenklinik, GPR Klinikum Ruesselsheim, Ruesselsheim; Abteilung Kinder und Jugendliche & Abteilung Geburtshilfe, Dr. Horst Schmidt Kliniken, Wiesbaden; Kinderklinik & Klinik fuer Frauenheilkunde, Universitaetsklinikum des Saarlandes, Homburg/Saar; Kinderklinik & Klinik fuer Frauenheilkunde, Klinikum Saarbruecken, Saarbruecken.

**Italy (Emilia Romagna)**: Azienda Ospedaliero-universitaria di Modena, Modena; Azienda Ospedaliero-universitaria di Bologna, Bologna; Ospedale Maggiore C.A. Pizzardi, Bologna; Azienda Ospedaliera di Reggio Emilia, Reggio Emilia; Azienda Ospedaliero-universitaria di Parma, Parma; Ospedale Infermi, Rimini; Ospedale M. Bufalini, Cesena; Azienda Ospedaliero-universitaria di Ferrara, Ferrara; Ospedale Santa Maria delle Croci, Ravenna; Ospedale Morgagni – Pierantoni, Forlì; Ospedale Guglielmo da Saliceto, Piacenza; Ospedale Civile Nuovo Santa Maria della Scaletta, Imola; Ospedale Degli Infermi, Faenza; Ospedale Santa Maria Bianca, Mirandola; Ospedale Civile Guastalla, Guastalla; Ospedale SS. Annunziata, Cento; Ospedale Umberto I, Lugo; Ospedale Unico della Val D’Arda, Fiorenzuola D’Arda; Ospedale San Secondo, Fidenza; Ospedale B. Ramazzini, Carpi; Ospedale Pavullo nel Frignano, Pavullo nel Frignano; Nuovo Ospedale Civile di Sassuolo, Sassuolo; Ospedale di Bentivoglio, Bentivoglio.

**Italy (Lazio Region):** Policlinico Umberto I; Policlinico A. Gemelli; Azienda Ospedaliera San Camillo; Azienda Ospedaliera San Giovanni; Azienda Ospedaliera San Filippo Neri; Ospedale Pediatrico Bambino Gesù; Ospedale Belcolle-Viterbo; Ospedale Sant’Eugenio; Ospedale SG Calibita Fatebenefratelli; Ospedale S. Pietro Fatebenefratelli; Policlinico Casilino.

**Italy (Marche):** Azienda Ospedaliero-universitaria Ospedali Riuniti Umberto I – G.M. Lancisi – G. Salesi, Ancona; Ospedale Generale Provinciale C.G. Mazzoni, Ascoli Piceno; Ospedale Generale Provinciale Macerata, Macerata; Azienda Ospedaliera San Salvatore, Pesaro; Ospedale A. Murri, Fermo; Ospedali Riuniti di Jesi, Jesi; Ospedale Civile E. Profili, Fabriano; Ospedale Santa Croce, Fano.

**The Netherlands (Eastern & Central):**

Radboudumc Nijmegen; Wilhelmina Childrens Hospital Utrecht; Canisius Wilhelmina Hospital Nijmegen; Maasziekenhuis Pantein Boxmeer; Bernhoven Hospital Uden; Gelderse Vallei Hospital Ede; Rijnstate Hospital Zevenaar; Rijnstate Hospital Arnhem; Slingeland Hospital Doetinchem; Regional Hospital Koningin Beatrix Winterswijk; Gelre Hospital Zutphen; Meander Medical Center Amersfoort; Gelre Hospital Apeldoorn; Diakonessenhuis Utrecht; Zuwe Hofpoort Hospital Woerden; Tergooi Hilversum; Hospital Rivierenland Tiel; St Antonius Hospital Nieuwegein; TweeSteden Hospital Tilburg; St Elisabeth Hospital Tilburg; Deventer Hospital Deventer; Tergooi Hospital Blaricum.

**Poland:** Department of Neonatology, Poznan University of Medical Sciences (III level); Szpital Wojewódzki w Poznaniu (II level); Szpital Miejski im. Franciszka Raszei w Poznaniu (II level); Specjalistyczny Zespół Opieki Zdrowotnej nad Matką i Dzieckiem w Poznaniu, Szpital Św. Rodziny (II level); Wojewódzki Szpital Zespolony im. L. Perzyny w Kaliszu (II level); Wojewódzki Szpital Zespolony w Koninie (II level); Wojewódzki Szpital Zespolony w Lesznie (II level); Szpital Specjalistyczny im. Stanisława Staszica w Pile (II level); Zespół Zakładów Opieki Zdrowotnej w Ostrowie Wielkopolskim (III level); Szpital Powiatowy im. Prof. Romana Drewsa w Chodzieży (I level); Zespół Zakładów Opieki Zdrowotnej w Czarnkowie (I level); Samodzielny Publiczny Zespół Opieki Zdrowotnej w Gostyniu (I level); Zespół Opieki Zdrowotnej w Gnieźnie (I level); Samodzielny Publiczny Zakład Opieki Zdrowotnej w Grodzisku Wielkopolskim (I level); Zespół Zakładów Opieki Zdrowotnej w Jarocinie (I level); Samodzielny Publiczny Zakład Opieki Zdrowotnej w Kępnie (I level); Samodzielny Publiczny Zakład Opieki Zdrowotnej w Kole (I level); Samodzielny Publiczny Zakład Opieki Zdrowotnej w Kościanie (I level); Samodzielny Publiczny Zakład Opieki Zdrowotnej w Krotoszynie (I level); Samodzielny Publiczny Zakład Opieki Zdrowotnej im. K. Hołygi w Nowym Tomyślu (I level); Samodzielny Publiczny Zakład Opieki Zdrowotnej w Obornikach (I level); Zespół Zakładów Opieki Zdrowotnej w Ostrzeszowie (I level); Pleszewskie Centrum Medyczne w Pleszewie (II level); Szpital Powiatowy w Rawiczu (I level); Samodzielny Publiczny Zakład Opieki Zdrowotnej w Międzychodzie (I level); Samodzielny Publiczny Zakład Opieki Zdrowotnej w Słupcy (I level); Szpital w Śremie (I level); Samodzielny Publiczny Zakład Opieki Zdrowotnej im. Dr J. Dietla w Środzie Wielkopolskiej (I level); Samodzielny Publiczny Zakład Opieki Zdrowotnej w Szamotułach (I level); Szpital Powiatowy im. Jana Pawła II w Trzciance (I level); Samodzielny Publiczny Zakład Opieki Zdrowotnej w Turku (I level); Zespół Opieki Zdrowotnej w Wągrowcu (I level); Samodzielny Publiczny Zakład Opieki Zdrowotnej w Wolsztynie (I level); Szpital Powiatowy we Wrześni (I level); Szpital Powiatowy w Wyrzysku (I level); Szpital Powiatowy im. A. Sokołowskiego w Złotowie (I level).

**Portugal (Northern Region)**; Centro Hospitalar de Entre o Douro e Vouga, E.P.E. - Hospital de São Sebastião; Centro Hospitalar de Trás-os-Montes e Alto Douro, E.P.E. - Hospital São Pedro; Centro Hospitalar de Vila Nova de Gaia/Espinho, E.P.E. - Unidade II; Centro Hospitalar do Alto Ave, E.P.E. - Unidade de Guimarães; Centro Hospitalar do Médio Ave, E.P.E. - Unidade de Famalicão; Centro Hospitalar do Porto, E.P.E. - Maternidade Júlio Dinis; Centro Hospitalar do Tâmega e Sousa, E.P.E. - Hospital Padre Américo; Centro Hospitalar Póvoa de Varzim - Vila do Conde, E.P.E. - Unidade da Póvoa de Varzim; Centro Hospitalar São João, E.P.E. - Hospital São João; Hospital de Braga; Unidade Local de Saúde de Matosinhos, E.P.E. - Hospital Pedro Hispano; Unidade Local de Saúde do Alto Minho, E.P.E. - Hospital de Santa Luzia; Unidade Local de Saúde do Nordeste, E.P.E. - Unidade de Bragança.

**Portugal (Lisbon and Tagus Valley Region)** : Centro Hospitalar Barreiro Montijo, E.P.E.- Hospital Nossa Senhora do Rosário; Centro Hospitalar de Lisboa Central, E.P.E. - Hospital Dona Estefânia; Centro Hospitalar de Lisboa Central, E.P.E. - Maternidade Alfredo da Costa; Centro Hospitalar de Lisboa Ocidental, E.P.E. - Hospital de São Francisco Xavier; Centro Hospitalar de Setúbal, E.P.E. - Hospital São Bernardo; Centro Hospitalar do Médio Tejo, E.P.E. - Hospital Doutor Manoel Constâncio; Centro Hospitalar do Oeste - Unidade de Caldas da Rainha; Centro Hospitalar do Oeste - Unidade de Torres Vedras; Centro Hospitalar Lisboa Norte, E.P.E. - Hospital Santa Maria; Hospital Cuf Descobertas; Hospital da Luz; Hospital de Cascais Dr. José de Almeida; Hospital de Santarém, E.P.E.; Hospital Garcia de Orta, E.P.E.; Hospital Lusíadas Lisboa; Hospital Professor Doutor Fernando Fonseca, E.P.E.; Hospital Vila Franca de Xira.

**Sweden (Stockholm):** Department of Obstetrics and Gynecology, Danderyd Hospital, Stockholm; BB Stockholm AB, Danderyd Hospital, Stockholm; Department of Obstetrics and Gynecology, Karolinska University Hospital (units in Solna and Huddinge), Stockholm; Department of Obstetrics and Gynecology, Sodersjukhuset (Stockholm South General Hospital), Stockholm; Department of Obstetrics and Gynecology, Sodertalje Hospital; Department of Neonatal Medicine, Karolinska University Hospital (units in Danderyd, Solna and Huddinge), Stockholm; Sachs' Children and Youth Hospital, Stockholm.

**United Kingdom (Northern Region):** Royal Victoria Infirmary Newcastle upon Tyne; James Cook University Hospital Middlesbrough; North Tees University Hospital Stockton; Sunderland Royal Hospital; Wansbeck Hospital Ashington; Queen Elizabeth Hospital Gateshead; North Tyneside General Hospital; South Tyneside General Hospital; Cumberland Infirmary Carlisle; West Cumberland Infirmary.

**United Kingdom (East Midlands - Yorkshire & Humber):** Chesterfield Royal Hospital;Bassetlaw District General Hospital;Kings Mill Hospital;Royal Derby Hospital;Nottingham City Hospital;Nottingham Queen's Medical Centre;Lincoln County Hospital;Boston Pilgrim Hospital;University Hospitals of Leicester (LGH + LRI);Kettering General Hospital;Northampton General Hospital;Grimsby Diana Princess of Wales Hospital;Scunthorpe General Hospital;Barnsley District General Hospital;Rotherham District General Hospital;Doncaster Royal Infirmary;Jessop Wing Sheffield;Airedale District General Hospital;Bradford Royal Infirmary;Dewsbury District General Hospital;Halifax Calderdale Royal Infirmary;Harrogate District General Hospital;Hull Royal Infirmary;Leeds General Infirmary; Leeds St James's;Scarborough District General Hospital;York District Hospital;Wakefield Pinderfields General Hospital.
